# Lockdowns and Physical Activities: Sports in the Time of COVID

**DOI:** 10.3390/ijerph19042175

**Published:** 2022-02-15

**Authors:** Mónika Harangi-Rákos, Christa Pfau, Éva Bácsné Bába, Bence András Bács, Péter Miklós Kőmíves

**Affiliations:** 1 Institute of Rural Development, Regional Economy and Tourism Management, University of Debrecen, H-4032 Debrecen, Hungary; rakos.monika@econ.unideb.hu; 2 Institute of Sport Management, University of Debrecen, H-4032 Debrecen, Hungary; bacsne.baba.eva@econ.unideb.hu; 3 Institute of Accounting and Finance, University of Debrecen, H-4032 Debrecen, Hungary; bacs.bence.andras@econ.unideb.hu; 4 Károly Ihrig Doctoral School of Management and Business, University of Debrecen, H-4032 Debrecen, Hungary; 5 Institute of Economics and World Economy, University of Debrecen, H-4032 Debrecen, Hungary; komives.peter.miklos@econ.unideb.hu

**Keywords:** COVID-19, sports, physical activities, social classes, health preservation, aging, life expectancy

## Abstract

As aging causes challenges in several countries globally, more and more people are suffering from bad health conditions. Nowadays, COVID-19 causes many problems—and one of the root causes of these problems is the isolation of people from each other. The aim of our article is to investigate the effects of COVID-19 on people’s physical activity. Physical activity is strongly correlated with health status and health preservation is very important to minimize the negative effects of the pandemic. In order to investigate this topic, we prepared an extended literature review, citing the most important sources of COVID-related health-preservation issues. Our results showed that the negative economic effects of the coronavirus pandemic have caused a decrease in physical activities in several cases. A reduction in possible physical activities has a potential negative effect on the life expectancies of elderly people. In order to underline the importance of physical activities, we prepared an extended literature review, aiming to summarize the available knowledge related to COVID-19. As a conclusion we recommend to strengthen, maintain, and develop available sporting possibilities for people. The most important of these recommendations is the development of physical activities that are available for free.

## 1. Introduction

According to Nieman [1], the overall state of health of humankind is getting worse. As a result of the welcome increase in life expectancy, there are more countries in the world today in which the population is aging rapidly. There are also growing numbers of people in the world who lead a sedentary lifestyle, and, as a consequence, they suffer from various diseases or illnesses, such as obesity or diabetes. In turn, deteriorating health conditions facilitate recurring epidemics or even pandemics, such as the most recent one, prompted by the new coronavirus, also recognized as COVID-19 or SARS-CoV-2.

The objective of our study is to give an account of how the spread of this novel type of coronavirus has influenced the habits and customs of people in the context of doing sports. With the help of sports, health conditions may be improved, and an active lifestyle does indeed contribute to preserving health; however, due to the fact that the peculiarities of the transmission of this novel type of coronavirus, including direct droplet infection and infection by touching infected objects, allow for an extraordinarily rapid spread, and that the symptoms of the disease vary widely, from completely asymptomatic cases to fatal outcomes, and restrictive measures have been introduced in almost all countries around the world. These measures are particularly justified by the ability of asymptomatic patients to carry and spread the disease. Among the most frequently imposed restrictions, we highlight the following: mandatory hand disinfection, mandatory face and nose mask use in enclosed spaces (and later in open spaces), quarantine obligations by patients and those under examination, partial lockdowns and curfews, restrictions on the freedom of assembly, closing down schools and introducing distance education, the preference of digital working from home, and even border lockdowns, the consequence of which has been difficulty (and sometimes impossibility) in international travel and transportation. Although it is not necessarily interpreted as a restriction, people are also advised to increase physical distance between themselves and others, and to comply with what has been termed “social distancing” [2].

As a result of the measures listed above, in quite a number of countries around the world, masses of the population have been forced to live secluded in their homes for shorter or longer periods of time, even in cases where they were not infected. In several locations, sports facilities were closed down due to the significant risk of contagion through droplet infection from physical activities. However, sports remain necessary for healthy people, even during quarantines and curfews, which is why supply will sooner or later inevitably meet demand.

## 2. The Major Characteristic Features of the Novel Type of Coronavirus

One of the most challenging difficulties in the struggle with the novel coronavirus has been caused by the circumstance that the symptoms of the virus vary significantly from patient to patient. Quite a number of those who are infected carry the virus without exhibiting any symptoms whatsoever, which is why the number of latent patients, as well as of those who are identified as carriers (either too late or never), may be quite high [3]. The disease can also potentially lead to a fatal outcome. Globally, by 4 January 2022, there have been 290,959,019 confirmed cases of COVID-19, including 5,446,753 deaths, reported by the WHO [4]. Struyf et al. [3], in their publication on a synthesis of medical studies available on coronavirus, list a total of 27 different signs and symptoms related to the disease, which can be grouped into the following four categories: systemic, respiratory, gastrointestinal, and cardiovascular. Our knowledge is rather restricted concerning the signs and symptoms of COVID-19, and the symptoms and results are highly variable across studies, which is partly due to the great diversity and variance of occurrence of symptoms. The most frequently experienced and identified symptoms include the following: cough, sore throat, fever, fatigue, headache, and myalgia or arthralgia. Relatively frequent symptoms include diarrhea, loss of the sense of taste and smell, shortness of breath or difficulty in breathing, disorientation or confusion, and chest pain or pressure. Examining patients who had been infected with, and then recovered from, COVID-19 in Italy, Carfì et al. [5] found that, among infected patients with symptoms who had received medical treatment and agreed to undergo post-treatment monitoring, only one-eighth reported no symptoms after just two weeks following the initial appearance of symptoms. At the same time, almost one-third of these patients reported the persistence of one or two symptoms, and more than half of them reported the persistence of at least three symptoms. In the case of about 44% of the patients, a worsened quality of life was reported due to infection with coronavirus. Among the most frequent symptoms reported to be persisting, even after the infection with COVID-19 ended, patients identified fatigue, dyspnea, joint pain, chest pain, cough, anosmia, dryness and irritation of the eyes, and rhinitis-like symptoms.

Ali and Alharbi [6] emphasized that patients infected with COVID-19 should, by all means, follow medical therapeutic prescriptions. In addition, all persons should follow preventive measures, management, and quarantine, which have been introduced, including curfew, social distancing, and hygienic regulations.

The restricted and oftentimes contradictory nature of the available knowledge concerning COVID-19 may well be exemplified by the examination of the meteorological or weather-related factors influencing the spread of the disease, which tend to display differences in fundamental significance, even in articles published in the same periodicals. Lin et al. [7], in their meteorological investigations related to the spread of COVID-19, stated that there was a negative correlation with maximum temperature. According to findings from the same research, a high relative humidity with a low temperature increases COVID-19 transmission, while a high humidity with a high temperature reduces COVID-19 transmission. Zhu et al. [8] reported that, on the basis of findings from regions in South America, a high value of relative humidity displayed a negative correlation with how fast COVID-19 could spread in regions of high humidity. According to Şahin [9], among the highest correlations that can be observed between the spread of the novel coronavirus disease and factors of weather, it was meteorological variables, such as wind speed and temperature (over a period of 14 days) that exerted the highest influence. However, Briz-Redón and Serrano-Aroca [10] discarded the possibility of a significant correlation between the spread of COVID-19 and temperature. Nevertheless, Zambrano-Monserrate et al. [11] highlighted improving air quality among the indirect environmental impacts related to the spread of COVID-19, which obviously contributed to the achievement of positive effects concerning overall health conditions.

The spread of COVID-19 has definitely posed significant challenges for the healthcare systems of several countries. In Europe, in the spring of 2020, the epidemic situation quickly became critical in Italy, and the Italian government tried to respond by introducing strict confinement and movement restriction measures. In addition, in order to alleviate the shortage of professionals in the healthcare system, hospitals activated retired physicians and medical students, as well as residents, who were also recruited to be on duty. As greater percentages of patient care capacities that were available in the healthcare system had to be preserved for patients infected with COVID-19, treatments of patients not requiring immediate intervention were postponed or canceled. Part of the reason for this measure was to spare them from possible infection, while another part was to be able to use the capacities, thus freed, for fighting the coronavirus. As a part of this process, therapeutic treatments for cancer patients also had to be suspended or rescheduled, among other things [12].

Based on a broad range of analyses of mortality data related to the coronavirus, Elgar et al. [13] found that, in countries with higher levels of economic inequalities within society, or with deficiencies in some dimensions of social capital, mortality rates for COVID-19 were also higher. Social trust and belonging to groups are also associated with more deaths, possibly due to a greater degree of behavioral contagion and incongruence with physical distancing policies. Overall, it can be stated that countries struggling with greater degrees of social inequality require a more robust public health response and more resources to contain the spread of COVID-19.

In addition to introducing the restrictive measures listed above, containing the spread of COVID-19 could be achieved through the development of vaccinations against the disease and the implementation of vaccination campaigns influencing a significant part of the world’s population. At the time of writing this article, vaccinations are already taking place in a number of countries around the world. Currently, there are several kinds of vaccines in use that have been developed. The development of vaccines by researchers has been aided by the availability of information and samples, collected during the 2002 MERS coronavirus epidemic in the Near East [14]. In their study, Gostin et al. [15] identified groups of individuals on the basis of their relationship towards the issue of vaccination. According to this classification, people either wish to receive vaccination against COVID-19 as soon as possible; wait as long as possible before getting vaccinated; would get vaccinated only if their school or workplace required it; or categorically refrain from receiving the vaccine. The authors of this study also summarized what sort of relevance acceptance or rejection of vaccination may imply beyond the preservation of one’s health. Among the groups of individuals involved in common areas of activity, Gostin et al. [15] considered the prioritizing of physician and healthcare employee access to vaccinations as necessary because of their high degree of responsibility and risk of getting infected. In regards to the case of pupils and students in public and higher education institutions, in addition to reducing the risk of contracting the disease, it is also important that these young people should not infect or spread the virus to the members of other higher-risk groups. From the point of view of restarting the economy, the vaccination of pupils in grade schools is also important, as parents who need to take care of young children during the application of distance education would be absent from work and would not necessarily be able to work from home at all (or only to a minor extent). In addition, as a precondition for allowing personnel presence at work, serious consideration may need to be given by some employers regarding the mandatory vaccination of the employees in order to prevent the development of an epidemic outbreak. However, the issue of mandatory vaccination also concerns ethical and moral aspects. The demand for vaccination on the part of employers can, in fact, be easily understood. For example, in Germany, the involvement of meat and poultry processing plants in the development of super-spreading local outbreaks of the coronavirus epidemic has been verified [16,17,18].

Among the major health-related features characteristic of COVID-19, such as its spread via droplet transmission, the transmission-increasing effect of enclosed spaces and meteorological conditions, as well as the risks connected to air humidity, clearly represent circumstances that are also directly related to doing sports. In the light of the above, it can be concluded that studying the effects of COVID-19 on sports in general, and on doing sports in particular, represents an issue that is relevant and topical, not only concerning competitive sports, but also in the case of grassroots sports and leisure sport activities. The effects of COVID-19 on competitive sports and related sport events could be clearly experienced by almost all in 2020. Gallego et al. [19] discussed postponing of the Chinese Grand Prix of FIA Formula 1, while also mentioning the possibility of canceling the Olympic Games in Tokyo. Parnell et al. [20], however, could already take for granted the postponement the Tokyo Olympic Games and UEFA EURO 2020. Both of these major sport events were then held a year later than originally scheduled. As Shervani et al. [21] pointed out, any postponement to major world sport events, including the Olympic Games in Tokyo, is extremely costly. The extra expenses in the case of the Olympics had already amounted to more than 6 billion USD by the summer of 2020. In addition, it also directly affected the operation of a number of economic entities as well. For example, besides numerous companies related to tourism and catering, it also exerted an influence on the operation of media enterprises. Moreover, the preparation by professional athletes also became more difficult during the period of the spread of the virus, while holding and hosting preparatory competitions became equally uncertain and precarious. It must be emphasized though that both the International Olympic Committee, which was responsible for making the necessary arrangements for the Tokyo Olympic Games, and the UEFA, responsible for organizing EURO 2020, highlighted, in relation to the postponements, that, for them, the primary concern was the preservation and safeguarding of the health of the athletes, the organizers (involved directly or indirectly in the implementation of the competitions), and the fans, which is why they did their best to prevent the spread of the virus by all means [22,23]. At the same time, Parnell et al. [20] also pointed out in their publication that the implementation model scheduled originally for the 2020 European Football Championship, while taking advantage of the easy ways that borders could be crossed within the European Union, made it possible for several cities in a variety of countries in Europe to host individual football games, thus reducing the individual local costs related to the organization, but could be considered rather unfortunate from an epidemiological point of view. Related to the spread of COVID-19 at a majority of the locations, it was specifically restrictions on geographical mobility that emerged as a relatively efficient precautionary measure, which proved to be the least compatible with the teams, organizers, and spectators, traveling back and forth across Europe. However, the announcement issued by UEFA mandated only the postponement of the European Championship, while the system of implementation remained the same that which was planned before [23].

Although the significance of competitive sports should not be underestimated, in this study, we still intend to examine the effects of the spread of coronavirus on non-professional and non-competitive grassroots sports and amateur sport activities, practiced for recreation and for the preservation of health. The relationship between sports and COVID-19 is multi-combinational, since sports and a healthy diet, in general, are certainly capable of improving the health of individuals in the first place [24,25,26,27,28,29,30,31,32,33,34,35,36,37,38,39,40,41,42], for example, by reducing the risk of appearance of cardiovascular or metabolic diseases, osteoporosis, cancer, obesity, or diabetes [43]. However, with the advent of COVID-19, the circumstances and conditions related to participating in sports, which had been taken for granted previously, also underwent significant changes.

## 3. Social Position and Physical Activities

The emergence of the new coronavirus has influenced situations related to physical activities in two different ways. On the one hand, performing a number of different kinds of sports has become more difficult than before. For example, types of exercises or workout done in enclosed spaces definitely belong in this category. On the other hand, the economic recession caused by the spread of COVID-19 has also exerted an influence on the financial situation of individuals open to doing physical activities, which could, in turn, have an impact on their ability and affinity to do sports.

Bourdieu [44] claimed that, when examining sports, they need to be separated from “playing games” on the one hand and from various forms of physical exercise or gymnastics on the other hand. In this case, the truly significant criteria for sports include the elaborate systems of rules, in which a major part of their development was played by English elite institutions of education, attended by young members of the nobility and the rich middle class. Accordingly, the development of sports represents an evolutionary element in the framework of different forms of activity, regulated by strict systems of rules, that were developed from folk customs and games connected to major traditional agricultural festivities. This process is fairly similar to the series of changes and transformations, where classic dances, regulated by strict step combinations and choreographies, as well as protocol, developed from a variety of folk dances. Bourdieu [45] also highlighted the fact that the ideal of “fair play” in sports serves to train future elite leaders, since the idea of fair competition here is more important than victory by all means, which is described by him as a plebeian principle. From the aspect of the current analysis, we regard all forms of physical exercise relevant, thus expanding the scope of our examination to other forms of physical activities while respecting Bourdieu’s definition at the same time. Nevertheless, Bourdieu [46] also considered it imperative that the individual branches of sports should not be examined independently, but, rather, as parts of a great system of sports combined. In the complex system of sports, each and every component has its own place and value. Thus, we intend to continue our analysis accordingly; by considering physical activity and sports a potential—and appropriate—implementation for preserving one’s health.

In order to explore and describe the connections between the various layers of society and being engaged in sports or physical activities, it is necessary to establish clear definitions of social strata. However, there are no such clear-cut definitions available. Typically, social statuses are characterized by the presence or absence of certain privileges, and by access to various resources or the lack thereof. Correspondingly, we classify people as “individuals living in poverty” when meeting even basic necessities proves to be difficult for them. Fróna et al. [47] pointed out that nutrient-poor food consumption tends to increase in cases where we also find less favorable social conditions. People living in poverty are also characterized by social exclusion [48]. Among the poor and destitute, the extent of involvement in physical activities is typically low, which largely is due to the fact that a significant proportion of leisure activities cannot be pursued free of charge. Thus, those who live on low levels of income will, in this case, primarily spend money on bare essentials required for subsistence or survival and, as a rule, will not be able to spare money for expenses related to sports [49]. For improving the situation of groups lagging behind and participating in physical activities to a lesser extent, it is necessary to produce analyses that accurately explore their situations, which should then be used for creating plans for the increase of physical activities in the light of the above. This is also necessary in order to make sure that a broader and ever-growing circle of individuals can benefit from the positive health-related effects of physical exercise and activity [50]. Furthermore, it is also essential that the possibility of doing sports should be arranged with an eye to the needs or demands of individual groups, and that the various programs and facilities should be accessible to those concerned [51]. Looking at the relationship between poverty and sports or physical activities in a global context, we find that there are quite a few developing countries around the world where access to sports opportunities or physical activity by citizens is extremely limited. In addition, it can also be seen that, as a result of development in previously poor countries, the most frequently adopted habits or customs of consumption coming from affluent societies are those that tend to negatively affect the health status of the population. It is also characteristic of the developing countries that, with the exception of a relatively small elite, most members of the population cannot afford to have expenses beyond those that are absolutely necessary for direct subsistence [52]. Reyes [53] summed up the most frequent reasons for the measurable differences in the field of physical activities, and his list included the factors of income-bracket differences and social status, in addition to qualification, gender, and age. Accordingly, the highest rates of physical activities can be measured in the circle of young males who belong to higher levels of social strata and who have relatively higher income and a better educational background. A less favorable financial situation, belonging to a lower social class, lower degrees of education, older age, and the female gender, are typically factors that, when applicable, decrease the chances of involvement in physical activities.

The relationship between physical activities and social standing is fairly well known: those who are in a more favorable social position typically do more sports than those who are not [54]. There is also quite an array of circumstances that substantially and significantly influence whether or not specific individuals can have access to opportunities to do sports or a chance to be involved in physical activities during the course of their lifetimes. Such circumstances include low income, long working hours, and difficulties of access, which in turn might comprise, in addition to geographical disadvantages, a number of factors: issues like racism, religious, or cultural prohibitions, and even language barriers [55]. Nevertheless, it makes sense to parallel the cultural and integrative role of sports with all of the above. What is sometimes called the passive consumption of sports, i.e., watching sport events on television, as well as reading and/or discussing sports news in the family or with friends, are considerably frequent activities. If we add to this the fact that, from a variety of sources, including either printed or electronic media, the proportion of items related to sports is impressive, we might consider it justifiable to state that the general interest in sports is fairly broad [56]. Kola-Bezka [57], in her studies, focused on the role of sports in social integration. She contended that both active and passive participation in sports are able to remove the distinctions between social statuses. The reason for this is simply that, when involved in sports activities, people are equal to one another, regardless of their individual circumstances. In other words, they struggle shoulder to shoulder, together, to achieve the same objective while participating in various forms of physical activity. Meanwhile, a spirit of belonging and the affinity to form community both develop, in the same way as during passive sport consumption, such as when watching a football game. These real-life situations are completely fine for networking and establishing personal connections because of this shared and common interest, as a result of which, as time passes, these can also evolve into crucial events influencing social status [58]. These kinds of positive effects can be demonstrated, even cases where members of groups are on the periphery of society, such as refugees or immigrants [59], who otherwise face numerous difficulties during the course of their integration [60,61,62,63,64,65,66,67]. Sport event volunteering can also be similarly effective for the purpose of increasing one’s social capital [68], which can be attained by volunteers to span differences, even in social standing or status. However, this possibility often remains only theoretical because those who belong to a lower social class are oftentimes left out of the opportunity to volunteer [69].

Children’s commitment to engage in sports activities tends to be largely dependent on the attitude of their parents, their relationship with sports, and their socio-economic situation. In a household where there is a larger amount of sports equipment, such as bicycles and balls, children will be more likely to persist in pursuing sports seriously than in cases where such pieces of sport equipment are not available in the household [70]. The relationship between the financial situation of families and the physical activities of children was also analyzed and described by Flintoff and Fitzgerald [71]. At the same time, a survey by Gatouillat et al. [72] of French teenagers highlighted the fact that the role of the family in continuing to practice sports, or dropping out, is more often relevant only at a younger age for kids, and the role and importance of friends in maintaining commitment to the practice physical activities increases as they get older. However, it needs to be emphasized that the proportion of students focusing on their studies rather than on sports also grows as they get closer to their final exams at school, and their study workload increases. According to Sanatkhah [73], the affinity to participate in sports and the activities of people (as these relate to their bodies), as well as their self-management of the body is also substantially dependent on what sort of media content they find on various platforms related to this topic. Media content accessed and preferred by young people is considerably diverse. Among these, in an ideal case, we can also find those that are connected to sustainability and health promotion [74]. However, young people born after 1995 increasingly tend to reject traditional media content, produced and compiled by the editorial staff of classic media sources. For them, it becomes more and more important to be able to select and choose from their own the circle of content that they are curious about or find interesting, and to be able to consume these when it is ideal for them to do so [75]. At the same time, this means that younger audiences also acquire knowledge concerning public issues from a variety of sources of new media [76], and this also includes various forms physical activities and sports.

As life expectancy keeps growing globally and, as a consequence, the aging of societies in certain, typically developed, countries also becomes an acute problem, there is more and more attention being paid to the quality of life of aging and older individuals. A pertinent proposal also raises the issue of senior citizens’ own responsibilities in relation to their health status. There is increasing attention being paid to older adults who can actively take care of their health and age, thus sustaining a better quality of life, even at an advanced age, thanks to their prudence and due diligence [77]. Quality of life at old age is largely dependent on the prevention of diseases, e.g., if older people have had the opportunity to live a healthy life at a younger age. As long as health protection and the prevention of diseases prove to be successful in the long run, the growing number of elderly will not overburden the public health system [78]. Tokarski [79] pointed out that, in later life, the elderly do not participate in sports because they get less and less interested physical exercise and are happy without sports. They tend to get involved in other hobbies, despite the fact that the positive effects of physical exercises on health are widely recognized. In their overview to summarize the results of datasets concerning sport participation and physical activity by the elderly, Tischer et al. [80] contended that there were significant discrepancies in the databank, not only between countries, but also between individual surveys as well. In general, it seemed that the level of physical activity tended to decrease with age, and that the range in the kinds of sports being practiced changed. Moderate and more predictable exercises, such as gymnastics, hiking, or cycling, become more popular than games, such as ball games. Older people’s motivation towards physical activity also changed. While a significant proportion of younger people consider exercise and workout as a pastime or a kind of entertainment, this seemed to be much less true for older people. At the same time, social activities appear to be a motivating factor or an opportunity, which influence the level of openness to physical activities of elderly people who live alone. The extent of physical activity, and openness to it, are also influenced by health status and the social position of the elderly. The average level of physical activity in their case was also higher among the wealthy than among those who live in worse circumstances. However, when calculating or measuring the extent of physical activity, what belongs in this category also needs to be defined. If we regard certain forms of housekeeping, gardening, or transport, such as walking or cycling, as manifestations of physical activity, the activity rate among the elderly definitely improves. Gardening, for example, if it incorporates subsistence or backyard agricultural activities, can also be practiced by the elderly who live in rural areas and have only a limited access to sports. Thus, they do not only exercise, but also produce healthy food for themselves [81,82,83]. However, the social physical activity missed and desired by lonely elderly people can be especially dangerous, especially during the COVID-19 pandemic. If they get infected, the mortality rate amongst the elderly is higher, not least of which because they are more likely to suffer from underlying conditions or disease [84].

In times of economic crises, governments typically tend to introduce austerity measures, which may also decrease public spending on sports. This usually affects the members of different social layers to varying degrees. In general, however, it can be stated that the chances and opportunities for lower social status groups to participate in sports typically deteriorate as a result of these austerity measures. The negative influences affecting such groups can also occur indirectly; for example, through reductions in funding available to social organizations that would otherwise normally support them [85]. Nevertheless, Roberts [86] found that more affluent people, such as members of the middle class, were able to outweigh the effects of this decline in public spending on sports by increasing their tailored individual expenditures, even during periods of economic crises. According to Parnell et al. [87], economic crises also exert a direct influence on sports organizations, and not only due to cuts in state or government subsidies. In times of crises, even fundraising routines that were used successfully previously can only be applied to a limited extent. The reasons for this are that the funds available to organizations supporting sports activities shrink on the one hand, while, on the other hand, the scope for the financial involvement of private individuals narrows equally. Nevertheless, or even in spite of all this, the expectations aroused concerning said organizations oftentimes do not take the limited opportunities left available due to the shifting economic circumstances into account. As a consequence, not only grassroots sport organizations, but also national sport federations, are forced to reconsider and redesign their strategies. As Giannoulakis et al. [88] also pointed out, for example, in Greece, as a result of the extreme economic slump after 2008 and the severe budgetary constraints following 2010, national sports federations also reported decreasing funding resources and, consequently, deteriorating conditions. In their view, due to declining state subsidies, they had to identify and introduce priorities in their operations and collect fees for services that used to be available free of charge to competitive athletes and they also needed to restructure their overall operation in general. As a result of these changes, the success ratio of Greece in several branches of sports declined.

Because of the spread of the novel coronavirus disease, the financial situation of numerous families around the world has changed, resulting typically in an increase in uncertainty and a decrease in income. For example, the closing down of schools during the spring of 2020, almost all over the world, had a significant economic impact. The objective of governments and competent epidemiological authorities was to prevent further spread of the disease. Up to one and a half billion pupils and students around the world were forced to leave their desks behind and to continue to study from home, if they were lucky. While this was not a problem for the parents of high-school and college students, parents of grade-school kids had to find a way to supervise their children at home or stay home as well, at least some of the time. As a consequence, parents who could not work from home had to take paid holidays and, after a while, unpaid holidays, while the risk of losing their jobs grew substantially [89]. The number of those receiving various benefits and allowances among employees who could not work from home office rose, since one of the most effective apparent solutions introduced for preventing the spread of the virus was social distancing. Thus, quite a few employers had to suspend their operations if social distancing was not possible at the workplace [90]. Obviously, not all jobs were affected in the same way by the economic effects of the spread of the novel coronavirus. Yet, the chances of losing one’s job in the sectors of trade and commerce, tourism, and catering increased significantly [91]. The spread of the new coronavirus also put low-wage workers in a particularly difficult position. The kind of work they perform is typically monotonous manual activity, during the course of which physical contact between individuals is quite frequent. In addition, low-income workers typically work in poorer working conditions. In some countries, health insurance allows them to only access a much narrower range of benefits. It is also possible that wage-based health insurance is not even available to them at all. A significant portion of low-income work is paid on a piece-rate basis, which means that no income is earned if an employee is absent. This poses an very complex dilemma for employees: they cannot afford to stay at home and work from there, while going to work and, therefore, coming into physical contact with other members of the community represents a concrete health risk, while the expenses incurred after potential infection exceed their available financial means. In view of the circumstances listed above, it can even be assumed that the mental health of low-wage workers might be at greater risk due to COVID-19 [92]. Furthermore, the spread of the novel coronavirus may put older employees at greater risk if they cannot successfully adapt to changing demands, requiring increased IT skills [93]. All in all, the least amount of economic disadvantages for employees was represented by working from home but, at the same time, this kind of remote work, or teleworking, was accompanied by an increase in the proportion of sedentary work [94], the health disadvantages of which are proven facts.

As a result of the spread of the novel coronavirus and of the measures taken to slow it down, the structure of household expenditures has also changed substantially. Individual households typically seek to reduce spending due to fears of uncertainty. There have been certain circumstances that have aided them in this endeavor: for example, a significant cut in transportation or commuting costs could be achieved because of working from home. The decrease in expenditures has also affected sports. On the one hand, levels of passive sport consumption went down as fans who purchased tickets to canceled games or games played behind closed doors only did so out of charity, with the intent of supporting their favorite sports clubs. In order to prevent the spread of the novel coronavirus, authorities and decision makers decided to close down sports clubs and sports venues in several locations, so the expenses of premium physical activities and workouts were also removed from household expenditures [95]. However, the specific way in which curfew hours and related restrictions were introduced in the United Kingdom during the spring of 2020 offered a chance for a relatively large proportion of the population to remain physically active, even during the pandemic. The regulations introduced there allowed non-quarantined citizens to leave their homes for 60 min every day in order to do some kind of physical exercise. During the course of being engaged in physical activity, they obviously had to comply with the epidemiological precautions and regulations, such as social distancing and public gathering restrictions, yet the opportunity to leave their homes legally prompted many people to exercise. Although physical activity was still low among people of lower social statuses, older adults, for example, were more willing to engage in exercise [96].

According to a cross-sectional representative survey related to the first and second waves of the COVID-19 pandemic in Hungary, the number of those that never do sports increased significantly. The figure before the pandemic was 64.17%, while during the course of the first wave, it went up to 78.33% [97]; during the course of the second wave, it was 73.67%. Although the situation was somewhat more favorable during the second wave than during the first, the decline was still significant. Despite some increase in the number of those participating in online sports (2.9% of all the athletes), this kind of practice and training did not become widespread [98].

Despite the fact that free exercise was available, even when curfew and confinement restrictions were in effect, a decrease in physical activity by the population was reported in quite a few places. In Hungary, as compared to the months prior to COVID-19, the physical activity of the population decreased by half an hour per week on average, which amounted to more than 30% (32%) [98].

Parallel with this, and partly in order to alleviate the depression and the fears caused by the spread of the pandemic, many people got accustomed to consuming foods that contain sugar in large quantities. These processed food varieties contain little in the way of valuable nutrients but a lot of energy; however, the human body—especially because of this easily accessible energy—is “happy” to welcome the consumption of these foods. As a result of the changing dietary habits, the decrease in physical activity, confinement during curfew, and, indirectly, because of the spread of the coronavirus, the level of the obesity in the population increased at a number of places [99]. All this must also be examined in the light of the fact that Hall et al. [100] went as far as describing the globally observable physical inactivity and the widespread sedentary way of life as another distinct epidemic, spreading in parallel with the novel coronavirus disease. This approach may be a bit exaggerated, although it is perhaps not without any grounds, given the risks and hazards of physical inactivity.

If we also take into account the fact that the spread of the novel coronavirus led to closures and lockdowns, which caused an economic recession that, in turn, increased unemployment and forced a great number of employees to take unpaid leave from work, it becomes clear that household expenditures are dominated by absolutely essential items. In situations when, even in developed countries, the ability of masses of people to cover basic expenses, such as rent, becomes questionable, the consideration of unnecessary items may easily disappear from family budgets [101]. Expenditures related to sports and physical activities decreased significantly with the advent of the novel coronavirus [102]. Nevertheless, lack of exercise due to confinement and the loneliness caused by isolation motivated quite a few people to find and apply alternative solutions to take care of their physical activity needs. Today, thanks to advancements in technology, there are numerous solutions available, not only for keeping in touch, but also for self-education in the field of physical exercise. In the next part of our study, we will present several versions of these possibilities, bearing in mind specifically the opinions of Oren et al. [103], who believe that the number of patients diagnosed with cardio-metabolic diseases may increase in the long run as a consequence of the restrictions on free movement due to the novel coronavirus, as well as from the lack of physical exercise and from the stress caused by the pandemic.

## 4. The Most Recent Solutions for Physical Activities Prompted by the Novel Coronavirus

The importance of staying physically active during the pandemic and lockdowns may be challenging for people, but it cannot be questioned. COVID-19 has an effect on the average level of physical activity and has a more serious effect on the elderly age group. Physical activity can make the health status of people better and the positive health effects can help prevent serious, possibly tragic outcomes of the illness. However, this information has been widely transmitted to citizens of several countries, and, in many cases, different population groups reduced their physical activity levels during COVID-19 lockdowns [104]. Clemente-Suárez et al. [105] recommended that people follow a physically active lifestyle during the pandemic and during lockdowns, with special regards to the legal prescriptions and recommendations, like social distance. The recommended level of physical activity is 150 min of moderate or 75 min of intensive physical activity, every 3–5 days, which can have a positive effect on personal health status. When gyms are closed due to lockdowns, it can be challenging for people to organize physical activities, but very simple and easily accessible tools are also suitable for physical activities, like climbing stairs, walking, running, or doing exercise. During lockdowns an increase of sedentary behavior has been reported in the case of all investigated population groups, including healthy adults, children, adolescents, and these same age groups suffering from any illnesses [106]. As Bentlage et al. [107] summarized, international and national healthcare-related organizations, like the World Health Organization or the American Heart Association, also underlined the importance of physical activity during lockdown periods related to the pandemic. It is important to respect all the preventive regulations, but, if possible, people should do some physical activity in the daytime. If regulations allow it, walking, jogging, and other isolated outdoor physical activities are able to cover prevention needs, and can have a positive impact on health. It is also important to transfer traditional physical activities to household duties: a good example of this was when the American Heart Association published that, from the viewpoint of physical activity, 20 min of vacuuming is equal to a one-mile-long walk. Rossi et al. [108] investigated changes in physical activities in the case of children and adolescents. Their results showed that COVID-19 has had a serious impact on the physical activities of these groups. Most organized sporting possibilities have been cancelled due to lockdowns during the different phases of the pandemic. The participation rates in unorganized sporting activities—like different outdoor activities and outdoor playing—increased, because most children and adolescents need to be physically active. The possibilities of physical activity in the case of children and adolescents mostly depend on their—and their families’—social status. Those who live in, for example, semidetached houses had a greater possibility to do physical exercises or play games outdoors than those living in apartment buildings or in a blockhouse. This trend meets the historical experiences regarding the correlations between fiscal status and sporting possibilities [109].

The novel coronavirus has also caused significant psychological problems for several population groups. Stress, anxiety, and mental distress are only three of the various illnesses possibly caused (or made more serious) by COVID-19. Disease incidence can depend on several factors, including age, labor status, living conditions, or other social factors [110]. Schoofs et al. [111] pointed out that several personal circumstances can affect physical activity levels. For example, in case of unemployment or COVID-related occupational changes, the average occurrence risk of a decrease in physical activities increased. Some other symptoms also emerged in the everyday lives of professional athletes during COVID-19. Tension, fatigue, and insomnia were typical in the case of athletes, but these health problems also appeared in the non-athlete population as well. Lockdowns and re-openings have had an impact on the overall quality of life; after the lockdowns, even the partial re-opening had a positive impact on the personal quality of life [112]. The prevalence of sleep problems and the risk of the development of different eating disorders also increased during lockdowns, while overall feelings regarding personal wellbeing and the impressions of quality of life decreased due to lockdowns [113]. In the case of Italian medical students, an increase in sleeping time was reported alongside with increased sedentary behavior, while a decrease of physical activities was also typical during the period of very strict lockdown regulations [114]. In another article, increased alcohol intake was reported during COVID-19 lockdowns, combined with less physical activity and more sedentary behavior in the case of university students [115]. Other negative dietary outcomes was observed in case of a French population sample, where the intake of snacks, sweets, chocolate, and cakes increased, while fresh fruit and vegetable intake decreased during the COVID-19 lockdowns [116]. On the other hand, we can also find some evidence for the correlations between physical activity and mental health. Those who could continue doing physical activity during the COVID-19 lockdowns, suffered less from different mental problems. The positive psychical outcomes of physical activity could have been realized during lockdowns and other quarantine situations [117].

As Elavarasan and Pugazhendhi [118] pointed out, digital solutions have become more broadly applied around the world as a result of the spread of the novel coronavirus. In accordance with the facts listed above, concerning how the spread of the virus can be slowed down through social distancing and reducing the number of person-to-person contacts, the use of the Internet and communication with the help of remote devices can be helpful in implementing a number of activities that previously required or postulated face-to-face encounters. Kelly et al. [119] also emphasized that as sports were created by no one other than us, the rules of the various branches of sports are not set in stone. Regarding COVID-19, this means that the rules that regulate sports can also be changed in order to make various forms of physical activity safer. As a matter of course, it has to be kept in mind that both athletes and fans expect individual branches of sport to be practiced according to well-established sets of rules, yet the need to protect participants’ health may have a strong enough legitimacy to allow for certain changes in regulations, even if only temporarily.

Smart phones and smartphone applications can indeed positively influence user behaviors and attitudes toward physical activity. Some smartphones monitor their user’s physical activity without the need to download any special applications. Thus, for example, they can track the number of steps taken and even send a polite warning message if the user is not physically active enough. In addition, there are thousands and thousands of free, and partially or fully premium, applications available for iOs and Android, the two major smartphone operating systems. In addition to general training programs, these include special software apps that prompt users to become more active by embedding, for example, running or jogging into a story frame or scenario. Communicating related individual achievements on social media is supported by most apps, which can consequently enhance amicable competition or rivalry among friends [120].

As modern technologies, such as smartphones, advance and their use becomes more widespread, novel forms of physical exercise can also be implemented. There is already a smartphone app that first assesses the user’s physique with the help of the built-in camera of the phone, compiles a professional training program, and then scores the user’s performance with the help of the same built-in camera, thus evaluating the exercise performed [121]. Some of these apps are not available for all types of smartphones, and, although certain packages of services offered are free of charge, there are also extra premium contents [122].

In their synthesizing study, Joseph et al. [123] gave a comprehensive review of how the Internet can be put to the service of physical activity. The communication channels and possibilities described in their paper, which basically summarized research methods applied in relation to physical activity, can be used in cases when an instructor would normally conduct conventional individual or group practices for participants, which are temporarily not possible in the traditional face-to-face format due to COVID-19. A common and frequently applied solution, for example, is to use Internet-based training and information modules that can be directed and controlled by the users themselves. In addition to assigning practical tasks, this solution also allows for the transfer of theoretical knowledge and even for the organization of said new knowledge into modules. Another fairly popular and recurrent solution is when computers provide feedback to users on the basis of incoming information inputs, the aim of which are to promote physical activity. In this case, software does not primarily analyze incoming images but, instead, compiles a custom-made proposal, tailored to improve physical activity, based on the responses to a questionnaire filled out by the user. As the third solution used by researchers to promote physical activity, instructors were accessible to users in a variety of chat rooms, where they can be directly asked questions in relation to physical activities. In this latter case, the contact established can also be complemented by sending email or other messages. The fourth appropriate solution can be when applications allow a user to set specific objectives and goals first, and then monitor their performance and provide feedback. In cases like this, the person involved in physical activity needs to try to achieve pre-determined goals, the results of which they receive as detailed feedback. Finally, for solution number five, it needs to be highlighted that online applications can provide social support to those engaged in physical activity. In this last case, physically active users can communicate, not just with the instructor, but with one another. Therefore, the flow of information, knowledge, and experience between users is possible, as is support and encouragement [123].

As digitalization in the world of sports had already been a tendency, regardless of the spread of the novel coronavirus, more and more sports organizations, including voluntary sports clubs, benefitted from reforms that were made possible by the digital switchover. This process has proved to be a shift affecting, not only the communication of organizations, but also their internal operations [124]. The benefits inherent in mobile communication, such as the continuous flow of information and uninterrupted accessibility, which are available if there is an appropriate Internet network, can significantly increase the efficiency of sports organizations. In addition, such organizations will be able to use the technology at their disposal more efficiently over time [125], which can prove to be an advantage during emergencies similar to the one caused by COVID-19. In addition, as technology advances, there will be even more special software and smartphone applications available in the future [126]. Thus, there will be more and more situations, also related to sports, the proper handling of which will have applications that are specifically designed for these concrete purposes [127].

A survey conducted with personal trainers in Norway showed that, not only had almost all of them lost some of their clients and could rely almost exclusively only on those who they had trained for a long time, but also that their individual and group online and outdoor sessions had multiplied. In spite of the latter effect, the living conditions of most personal trainers have ultimately deteriorated as a result of the spread of coronavirus [128]. In practice, this means that personal trainers are open to the use of modern technology, while their clients explicitly demand the availability of such sessions.

As a result of the spread of the novel coronavirus, professional sports came to a standstill for some time in almost all countries around the world. However, in order to maintain the health of the population, mass media outlets in the United Kingdom did their best to contribute to this effort by broadcasting special sports programs. In the framework of this endeavor, the best-known coaches of the UK conducted public training sessions, in which citizens that were open to physical activities could participate by watching the coverage. A similar role was taken by Britain’s most popular athletes, several of whom encouraged people to do sports through the communication tools available to them and provided them with expert advice on how to implement effective physical activity [129]. A tendency, which was very similar, was observed among celebrity influencers on Instagram, who were rather keen on posting content popularizing physical activity during the spread of the novel coronavirus [130].

The results of a cross-sectional survey conducted among Chinese students also showed that it is necessary to offer opportunities for sustainable physical activity during periods of voluntary isolation and official quarantine. In this context, social networks, videos, and targeted searches on information sites for health promotion are all appropriate tools [131]. Based on an analysis of an assessment of relevant international studies, López-Valenciano et al. [132] conclude that the level of physical activity among university students typically decreased as a result of the spread of the coronavirus and the consequent lockdowns. In several countries, this tendency developed because higher education switched to a distance education mode and used online classes, which—in the case of a high number of face-to-face contact hours—made it difficult for students to leave their home or residence. However, students who were involved in higher levels of physical activity before the pandemic continued to exercise as much as recommended, even if they did lower the level of their activity to some extent.

According to the findings of a survey conducted in German nursing homes, elderly patients living in such institutions were severely affected by COVID-19. Since institutions of this kind are particularly vulnerable to the spread of this disease, the primary objective there was to protect the life and health of the residents. Therefore, even the personnel responsible for ensuring the availability of proper physical exercise were not allowed to enter the homes. The reason for this was simply that, in the case of nursing homes, it was typically members of the staff that could potentially introduce the virus to the elderly from outside. In the absence of physiotherapists and other professionals, only nursing staff were able to provide limited assistance to maintain the required level of physical activity of the residents, so the physical and mental condition of the elderly began to deteriorate. In order to prevent this bad situation from escalating, physical education professionals were later allowed to resume their scheduled sessions, outdoors, where it was also possible to comply with the requirements of social distancing. However, isolation had taken its toll by then and resulted in a state where some of the elderly got tired more easily, with quite a few of them experiencing difficulties in even leaving their own rooms [133]. In any case, replacing indoor sessions with outdoor ones and conducting these in smaller groups of participants definitely turned out to be an innovative solution, prompted by the hazard of the spread of the novel coronavirus. A study by Brady et al. [134] also confirmed that memberships in fitness programs have a positive effect on the openness to physical activity and, consequently, to health preservation among the elderly. If there are several members joining such a program, the intensity of the experienced social advantages may increase. For older adults with serious mental illnesses, significant successes have been achieved through the application of computer games with gestural interfaces in the field of using digital platforms for physical activity. Group play through exergames for older adults has been found to promote recovery and healthy aging by significantly decreasing social isolation and increasing social integration [135].

Bentlage et al. [136], aiming at research synthesis through a systemic review of relevant articles, collected and classified tasks and good practices in a study about physical activity during the course of the spread of the novel coronavirus. In the first round of their recommendations, they discussed how to design programs related to physical activity. They underlined the importance of the fact that physical activity should be tailored specifically to the circumstances of home-based isolation. That is to say, exercises should be adapted to a home environment, for example, one’s apartment. Furthermore, they highlighted how, during the course of planning physical activity for home confinement, it is absolutely essential to bear in mind the importance of connection to the Internet, as well as to consider the daily routines of various groups. They gave the example of older adults, in whose case it was not enough to address them through the Internet in the first place. They also suggested that interventions of physical activity targeted at them should be scheduled for afternoon hours, when most elderly rest or demonstrate sedentary behavior. Their second set of proposals included examples of non-digital exercises and physical activity, which typically included tasks that can be performed indoors or in an enclosed space. One such example was an indoor gardening program, which aimed to increase the number of plants in the residential units to be taken care of. Another possibility that they considered good practice was Tai Chi. The third topic discussed focused on examples of physical activities performed with the help of digital technical solutions. Here, they underline that, by exploiting the opportunities offered by the Internet, it was not only possible to increase the level of physical activity, but also the frequency of social interactions, which can effectively improve the level of social integration. For example, they noted the advantages of joining virtual fitness programs and groups that, in addition to assisting in raising the level of physical activity, also facilitate the implementation of social interactions. The fourth batch of suggestions included good practices of so-called space-simulation isolation, the chief objective of which is to overcome and replace sedentary behavior or bed confinement. In this case, exercises help to resist the urge to minimalize physical activity. Finally, group five contained recommendations that served the purpose of transferring knowledge collected concerning so-called real-life isolation cases. Accordingly, it postulated the examination of groups of individuals that experience confinement in general, not necessarily due to a pandemic lockdown and, as a consequence, already have experience in processing the psychological burdens of isolation and loneliness [107].

Other researchers have thought about solutions for implementing physical activity that anyone can do at home. Da Cunha de Sá-Caputo et al. [136], for example, put together a test to assess one’s personal physical condition and monitor its development, in which there are also several workout activities that can be performed at home. These home-based exercises included: a stair climb test, balance test, single-leg-stance-test, gait speed, five-chair stand (checking the ability to sit down and stand up with arms folded), free walking, free run, six-minute walk test, timed up and go (measuring the ability of individuals to rise from a standard armchair, walk to a marker three meters away, turn, walk back and sit down again), sit-and-reach (measuring the flexibility of the lower back and hamstring muscles of individuals sitting on the floor), fingertip-to-floor test (measuring the ability of individuals to bend forward), and free physical exercises. It seems expedient to record and log the results of various tests in order to be able to properly monitor changes in one’s physical condition and physical abilities. Although the physical activities listed above may not seem particularly difficult or strenuous at first, with regard to the individual responsibility concerning the prevention of the spread of the novel coronavirus disease, suggestions that put less strain on people should also be presented and described. The reason for this is that, for people who are physically inactive, especially if they are overweight or obese, intense physical activity is not recommended since reactions of the body increase the risk of contracting the novel coronavirus in those cases. It seems that, as a consequence of moderate physical activity, the chances of the same effect occurring are significantly lower [137]. Ultimately, this means that, parallel with the strengthening of the body of previously inactive people, the physical load must be gradually increased in order to achieve optimal results that, in this case, would be an improvement in physical fitness and avoiding getting infected with the novel coronavirus disease.

At the time of the appearance of the novel coronavirus disease, there were quite a few modern technological solutions already available for maintaining and increasing physical activity and for avoiding loneliness, even in connection with physical activity. In addition to the examples described above, López-Valenciano et al. [132] also discussed the possibilities of gamification in their study. In their publication, based on international cooperation and extensive data collection from Asia, Africa, and Europe, Ammar et al. [138] found that people typically used the Internet more often following the outbreak of the pandemic than before. Among the reasons for increased use, satisfaction with communication needs was obviously present. From the aspect of the present study, more important than that, among the reasons for growing internet use, was the presence of meeting the needs for physical activity. Furthermore, people also used their social media platforms more extensively, as well as applications, smart watches, smart phones and fitness trackers at their disposal, in order to get involved in physical activity. This is reflected by the fact that, according to the health and fitness experts polled by the ACSM (American College of Sports Medicine), it was online training that became the new and most significant fitness trend in 2021 as a result of the pandemic [139].

Viana and Barbosa de Lira [140], by examining the benefits of exergame applications, which combine physical activity with the advantages of computer games, in coping with or tackling anxiety disorders that affected many during the course of the novel coronavirus, contended that, in addition to the benefits of maintaining physical activity, the available IT technologies also offered benefits in this area as well. Various applications proved to be especially successful, for example, in the field of Zumba or dance. Reporting on their survey conducted among university students in Wuhan, China, Deng et al. [141] presented several forms of physical activity, implemented through the application of web-based technologies. During these, the students could participate in events and opportunities hosted by the institution of higher education in a number of different ways: they could watch pre-recorded video clips, they could follow demonstrations of the exercises performed by the instructors live, and they could also communicate with instructors on the educational platforms available to them. The different kinds of sports, proposed by the institution to be practiced for the purpose of web-based physical activity, included shape-up exercises, designed combinations of exercises by teachers for a specific purpose, Chinese kung fu, rhythmic gymnastics, and table tennis. At the same time, students were sometimes confronted by technical problems when attending physical education classes and those who resided with their family during the lockdown, or moved home temporarily for the period of the pandemic, experienced difficulties in finding a suitable location for practice and exercises.

## 5. Conclusions

In 2020, with the global spread of the novel coronavirus, humankind experienced an emergency situation. The protection of human life and health suddenly became an overriding priority, while this inscrutable disease appeared in more and more places around the world.

Medical research on COVID-19 has revealed a wide variety of symptoms and potential courses of the disease. A significant portion of those infected can be asymptomatic during the entire course of infection, while others may very easily die as a consequence of it. Neither the onset of symptoms, nor the asymptomatic condition, have any correlation with infectivity, which means that asymptomatic individuals can also infect others involuntarily. For nearly a year, there was no vaccine available for the disease, so the best solution to slow the spread was the introduction of confinement restrictions and curfews. Patients contracting the disease were quarantined, while healthy individuals were encouraged to practice voluntary isolation.

As a result of the measures listed above, a global economic slump ensued, which severely affected national economies, employers, and masses of employees alike. In jobs where remote working was possible, working from home was allowed for a considerable number of employees around the world. However, workplaces where this was not a feasible solution were often forced to suspend their operations. Going to work was also hindered for employees by school closures that were implemented worldwide. At least one of the parents had to stay home to mind younger kids, oftentimes in the framework of unpaid leave. In this extraordinary situation, not even the grandparents were able to contribute to the supervision of their grandchildren, since the chances of a fatal outcome are more likely in cases when elderly people contract the virus. Therefore, a relatively stricter isolation of older adults and senior citizens was proposed all over the world.

The emerging economic crisis has pushed a huge number of families into varying degrees of existential crisis, which may end in another negative result in the form of a decrease in the level of physical activity. Restrictions on leaving the home, online work, and confinement have brought about an increase in the number of individuals who are physically inactive and lead a sedentary lifestyle, in addition to enhancing psychological problems, such as anxiety and depression. The latter effects would also be intensified in people simply because of the fear of disease. However, physical inactivity deteriorates human health, which increases the chances for a more serious course of disease in the case of infection. For certain groups, specifically including older adults, a decrease in the level of physical activity may lead to a sharp deterioration of health condition, resulting in fatigue or even the strengthening of mental problems as examples.

Nonetheless, the present development of information-communication technologies can alleviate or solve several of the related problems. With the help of telecommunication apps and software that are already available, people can stay in touch, which means that they can even do workouts together. It is also possible for coaches, athletes, and instructors of physical education to keep in touch with their clients and students in person, although virtually. As regards the results of practice, these can be shared on social media platforms, which may further strengthen motivation for keeping up with the activities. Applications available in the framework of gamification, as well as exergame applications, may multiply the number of positive experiences, making users more willing to exercise. Most of the available applications can be easily customized, which makes them perfect for individual target groups according to their health conditions or levels of fitness. Various liaison and contact management programs, such as conference systems, can also be used for the purposes of group-based physical activity.

Overall, maintaining physical activity may effectively contribute to reducing the negative effects of COVID-19. As long as people exercise or work out in compliance with the various regulations and restrictions, such as those regarding social distancing, they can be fitter and healthier and they can also break free of the monotonous routine of everyday life. All it takes is determination, and everyone can perform a variety of different exercises that are appropriate to their state of health.
